# Predicting treatment response using pharmacy register in migraine

**DOI:** 10.1186/s10194-019-0987-y

**Published:** 2019-04-02

**Authors:** Thomas Folkmann Hansen, Mona Ameri Chalmer, Thilde Marie Haspang, Lisette Kogelman, Jes Olesen

**Affiliations:** 1 Danish Headache Centre, Department of Neurological department, Copenhagen University Hospital, Nordreringvej 69, DK-2600 Glostrup, Denmark; 2 Novo Nordisk Foundation Center for Protein Research, Faculty of Health and Medical Sciences, University of Copenhagen, Copenhagen, Denmark; 3 Institute of Biological Psychiatry, Mental Health Centre Sct. Hans, Copenhagen University Hospital, Roskilde, Denmark

**Keywords:** Pharmacy database, Treatment response, Treatment predictors, Migraine

## Abstract

**Background:**

Precision medicine may offer new strategies to treat migraine, and access to existing large cohorts may be a key resource to increase statistical power. Treatment response data is not routinely collected for large cohorts; however, such information could be extracted from pharmacy databases. Using a clinical migraine sample with treatment effect data, we assessed whether treatment response can be predicted based on the number of drug purchases.

**Methods:**

A clinical cohort including 1913 migraineurs were interviewed using a semi-structured interview to retrieve treatment response data for acute and prophylactic migraine drugs. The purchase history was obtained from the Danish national pharmacy database. We assessed whether number of purchases at different thresholds could predict the specificity and sensitivity of treatment response.

**Results:**

Purchase history of drugs was significantly associated with treatment response. For triptan treatment the specificity and sensitivity were above 80% for individuals with at least ten purchases. For prophylactic treatment (beta-blockers, angiotensin II antagonists or antiepileptic) we observed a sensitivity and specificity above 80% and 50% for individuals purchasing any prophylactic drug at least four times. In the Danish pharmacy database, 73% of the migraine patients have purchased at least ten triptans, while 55–63% have purchased at least one of the four prophylactic drugs.

**Conclusion:**

Pharmacy databases are a valid source for identification of treatment response. Specifically for migraine drugs, we conclude that ten purchases of triptans or four purchases of prophylactic drugs are sufficient to predict a positive treatment response. Precision medicine may be accelerated with the use of pharmacy databases.

**Electronic supplementary material:**

The online version of this article (10.1186/s10194-019-0987-y) contains supplementary material, which is available to authorized users.

## Background

The success of precision medicine depends on the ability to identify patient groups with a specific response to a drug. The predictive power to classify patients depends on the quality and size of the initial cohort used to build the models [1]. Retrieving treatment response data for a large cohort is resource intensive and can take a long time. Identification of treatment response markers, e.g. in existing pharmacological databases that are easily accessible, is an alternative and simple strategy for assessing large cohorts. Although several countries maintain registry databases with information on individual medication use [2], this approach has never been tested. Migraine is an ideal condition to test the accuracy of prediction of treatment response from pharmacological databases. Migraine affects 15–20% of the population [3] and both acute and prophylactic treatments are available [4]. Triptans are migraine-specific acute drugs with no effect on peripheral pain [5] and are reported to be effective in 60–70% of treated migraine patients [6]. There are several (non-specific) prophylactic drugs for migraine available and the current choice of prophylactic treatment is made by trial and error [4, 7].

In Denmark, the national pharmacy database holds individual-level data on all prescriptions and subsequent purchases of drugs [8]. In a large clinical sample of migraine patients, we have collected information regarding migraine treatment response for both acute and prophylactic drugs [9]. We hypothesized that drug purchases can predict treatment response. To test this hypothesis, we combined the Danish pharmacy database with our clinical migraine sample and provide an estimate of the sensitivity and specificity to predict treatment response.

## Methods

Patients were recruited as part of the migraine genetic cohort at the Danish Headache Centre (tertiary headache referral centre) from 2010 to 2016. All patients were interviewed by medical doctors or senior medical students specifically trained in using a semi-structured interview to diagnose headache according to the International Classification of Headache Disorders (ICHD) [10]. A total of 1913 migraine patients with or without typical aura answered questions regarding medication use and treatment response to relevant headache pharmacological treatments including acute treatment (following categories: triptans (general, non-specific), ergotamine, non-migraine specific analgesics) and prophylactic treatment (following categories: Beta-blockers, Angiotensin II antagonists, Antiepileptics, ACE-inhibitors, and Anti-depressives). Acute treatment effect was considered positive if the patient reported at least 50% pain reduction within two hours of taking the drug. Prophylactic treatment was considered effective if the patient reported a reduction of at least 50% in the number of migraine attacks with three months of drug use. Patients who did not remember or had not tried the medication in question were registered as missing data.

The Danish medical prescription register is a national database in which all purchases of drugs prescribed by a medical doctor have been registered since 1994; data from 1994 until 2016 was included in the analysis. The register is primarily used for socioeconomic evaluation of medication use in Denmark. Prescription data for headache treatments often used in Denmark (Table 1) were merged at Denmark Statistics where study participants were fully anonymized. In Denmark, it is possible to buy non-migraine specific analgesics over the counter (OTC) and it is not possible to retrieve information about the purchase of non-migraine specific analgesics.

**Table 1:** Drugs assessed in pharmacy database

	Drug	ATC Code
Acute	Triptans	
	Sumatriptan	N02CC01
	Zolmitriptan	N02CC03
	Naratriptan	N02CC02
	Rizatriptan	N02CC04
	Almotriptan	N02CC05
	Eletriptan	N02CC06
	Frovatriptan	N02CC07
	Ergot alkaloids	
	Ergotamine	N02CA52
	Non-migraine specific analgesics	
	Paracetamol	N02BE01
	Treo	N02BA51
	Ibuprofen	M01AE01
	Naproxen	M01AE02
	Tolfenamsyre	M01AG02
	Diclofenac	M01AB05
Prophylactic	Beta-blocker	
	Metoprolol	C07AB02
	Propranolol	C07AA05
	Angiotensin II antagonist	
	Candesartancilexetil	C09CA06
	ACE Inhibitor	
	Lisinopril	C09AA03
	Antiepileptics	
	Topiramate	N03AX11
	Valproate	N03AG01
	Antidepressive	
	Amitriptyline	N06AA09
	Others	
	Pizotifen	N02CX01

### Statistics

We used R version 3.4.1 in RStudio version 2.1 for statistical analysis, with the R packages sas7bdat, ggplot2, caret, and ROCR. We created a confusion matrix for treatments reported with the effective measure in more than 5% (*n* = 100) of the assessed patients and used the confusion matrix function from the R package (caret to retrieve specificity, sensitivity and accuracy [11]. To test the influence of age, gender, and the interaction thereof, we used logistic regression to compare the contribution of each covariate towards the treatment effect and the influence on receiver operating characteristics (ROC). The number of purchases is given as median and quartiles as these data are not normally distributed.

## Results

A total of 1913 migraine patients were questioned about the use and effect of acute and prophylactic migraine treatments. Treatment effect of ACE-inhibitors and anti-depressive medication were reported by less than 5% and were only included in the combined analysis of all prophylactic treatments, see Table 2.

**Table 2:** Number of patients with or without an effect of migraine medication

		Treatment					
		All (n = 1913)		Males (*n* = 540)		Females (*n* = 1373)	
	Drug category	Yes	No	Yes	No	Yes	No
Acute	Triptans	1113	252	252	65	861	187
	Non-migraine specific analgesics	482	1080	159	288	323	792
	Ergotamine	89	132	23	21	66	111
Prophylactic	Beta-blocker	187	486	33	112	154	374
	Angiotensin II antagonists	232	325	47	73	185	252
	Antiepileptic	120	325	28	69	92	256
	ACE-inhibitor ^a^	1	21	1	14	–	7
	Anti-depressive ^a^	3	27	3	17	–	10

^a^Not analyzed individually

### Acute treatment

**Fig. 1:**
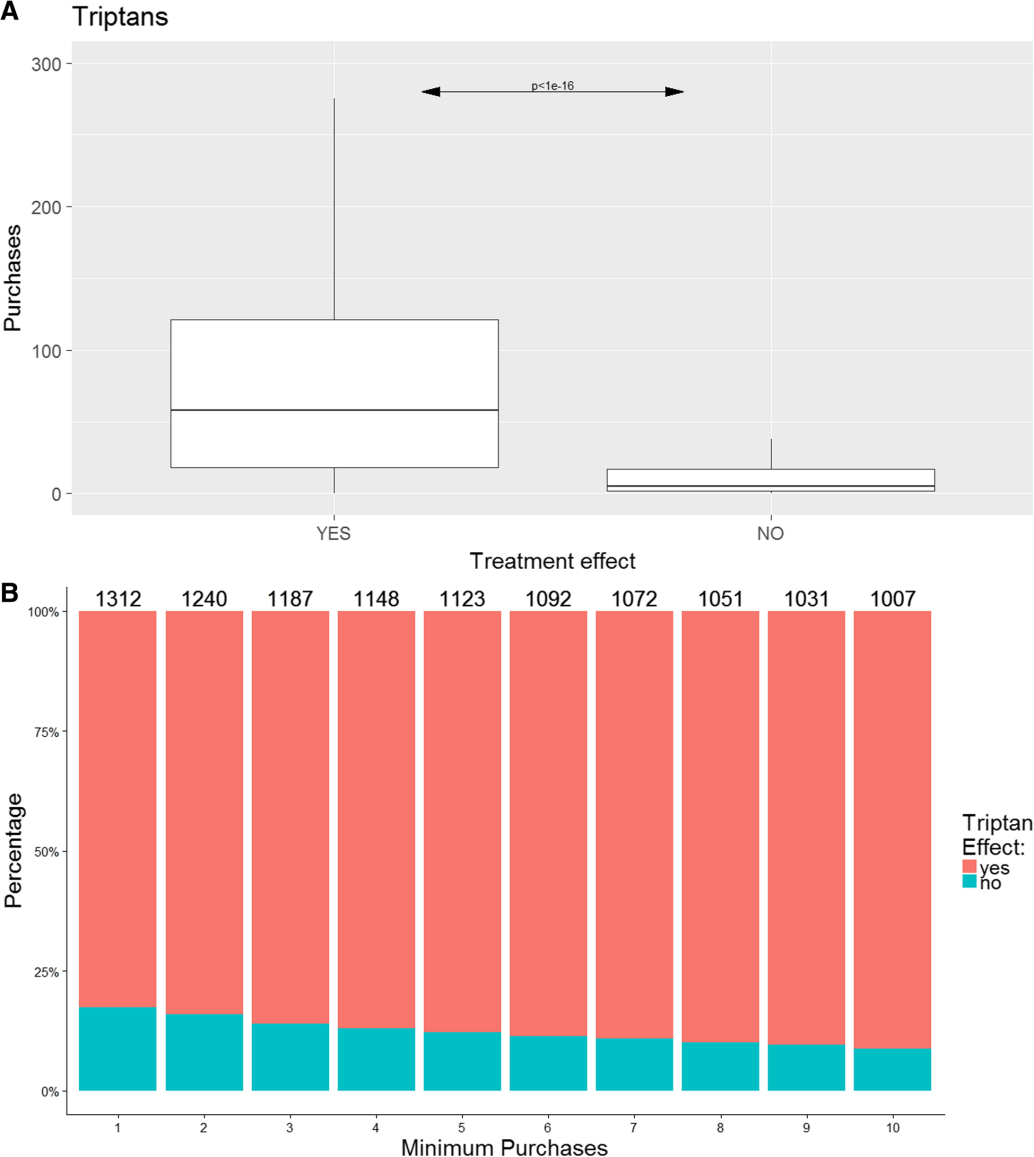
Distribution of triptan purchases. The figure depicts the distribution of triptan purchases, **a**) as a boxplot of individuals reporting a treatment effect of triptans (YES), no effect (NO) and **b**) as stacked histograms showing the distribution of triptan effect among patients with a minimum number of purchases of triptans on the x-axis and the total number of patients above each bar. Since the Danish legislation does not allow depicting individual data, outliers are excluded in **a**

**Fig. 2:**
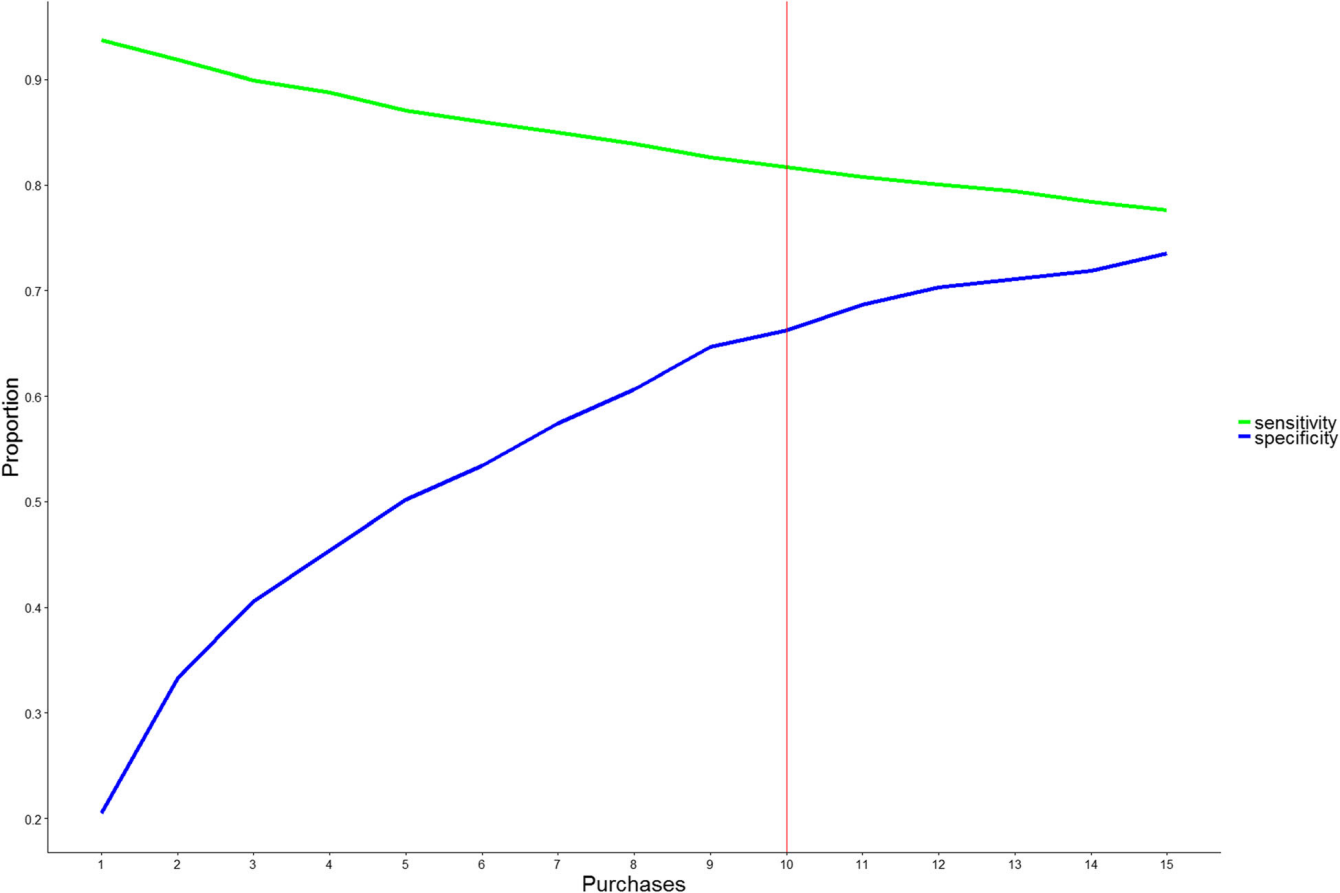
The sensitivity and specificity for triptan purchases to predict a positive treatment response. The figure presents the sensitivity (green) and specificity (blue) at different thresholds of minimum number of triptan-purchases, e.g. patients with ten or more triptan purchases had a sensitivity of 82% and specificity of 66% for prediction of positive treatment response (red line)

Increased number of purchases was significantly associated with a reporting of positive effect of any acute treatment (*p* < 1e-16). However, this signal was markedly driven by triptan purchase (Fig. 1), as both ergotamine and non-migraine specific analgesics did not differ regarding treatment effect (*p* > 0.05). Distribution of ergotamine and non-migraine specific analgesic acute treatments is presented in Additional file 1: Figure S1. Using logistic regression, we included age and gender but did not find a significant contribution to the ROC-curve, see Additional file 1: Figure S5. We calculated the sensitivity, specificity and accuracy at different thresholds for the number of purchases of triptans for prediction of a positive treatment response using a simple confusion matrix (Fig. 2). The overall sensitivity, i.e. the ability to identify a true positive response (Fig. 2 - green line), was above 80% at all triptan purchase thresholds analysed. However, the specificity, i.e. the ability to detect a true negative response, depended more on the number of purchases. Here, at least ten purchases were needed to gain 70% specificity (Fig. 2 - blue line). Here, 73% of the cohort purchased at least ten triptans (Fig. 1). The accuracy, i.e. the ability to predict overall true findings, was above 80% at all thresholds of purchases analysed. As triptans are known to have higher efficacy in migraine without aura than in migraine with aura, we repeated the analysis excluding migraine with aura patients [12]. Although reducing the statistical power, we found a generally better sensitivity but a lower specificity for migraine without aura, see Additional file 1: Figure S3.

In Denmark, it is clinical practice for neurologists to try at least three different triptans before concluding that triptans do not have an effect (personal communication prof. Jes Olesen). Thus, we tested whether having tried four or more different triptans could indicate a negative treatment response. We calculated a sensitivity of 21% and a specificity of 73% to predict a negative treatment response with an accuracy of 63%, see the distribution in Additional file 1: Figure S2. Furthermore, using the number of purchases we tested whether having only one prescription could predict negative treatment response. We found a sensitivity of 80% and a specificity of 6% with an accuracy of 20%. For the most prescribed drug, sumatriptan, we also analysed whether patients in monotherapy (*n* = 50) had different purchase number with a sensitivity of 80%. Noting that the number of patients in monotherapy was small, we observed that fewer, six purchases, gave the same results as for all triptans combined.

### Prophylactic treatment

**Fig. 3:**
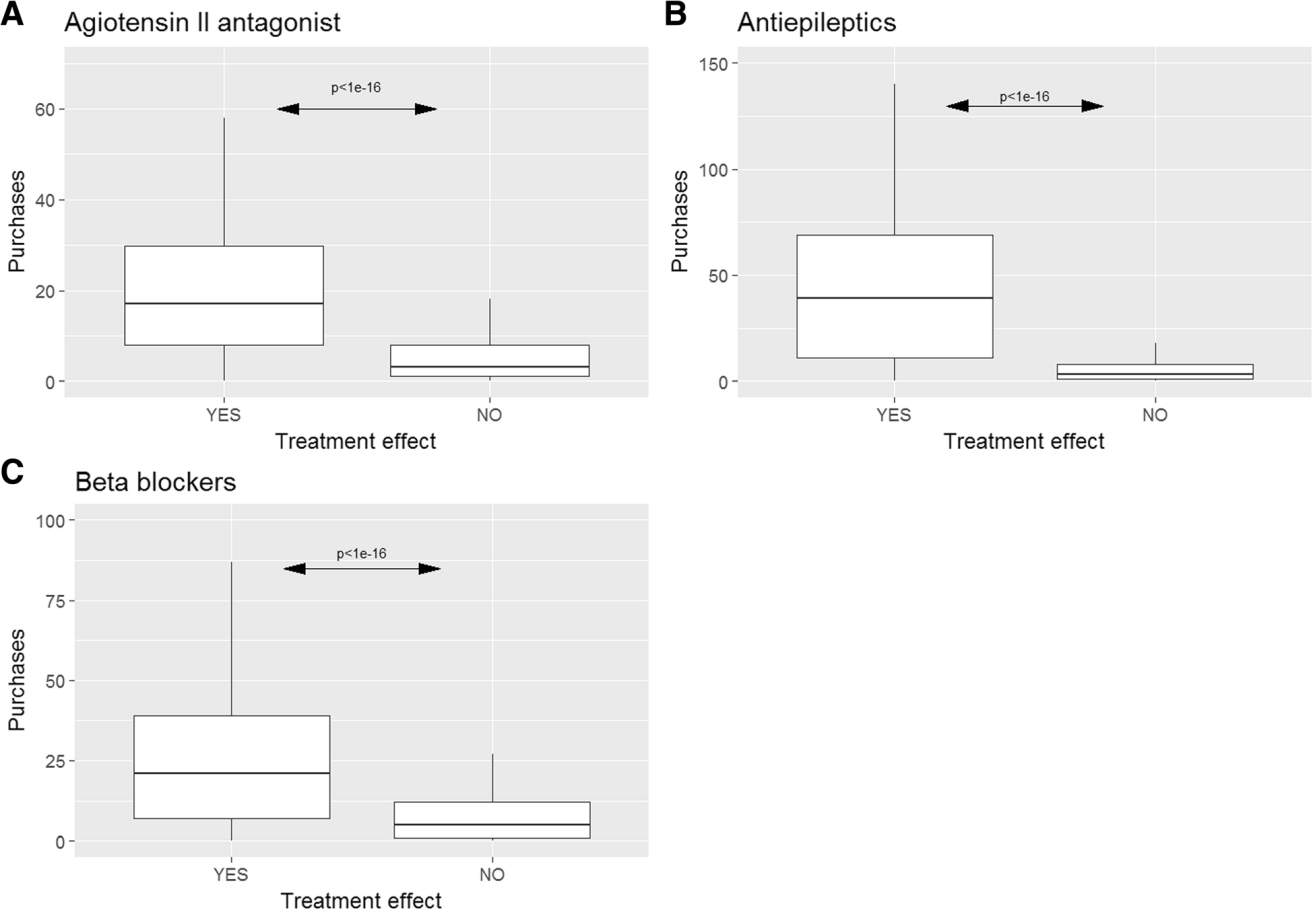
Distribution of purchases of prophylactic treatment. The figure depicts the distribution of **a**) Angiotensin II antagonist, **b**) Antiepileptic, and **c**) Beta-blockers prescription as a boxplot of patients reporting a treatment effect of triptans (Yes), or no effect (No). Given the Danish legislation it is not allowed to depict individual data, thus outliers are excluded

**Fig. 4:**
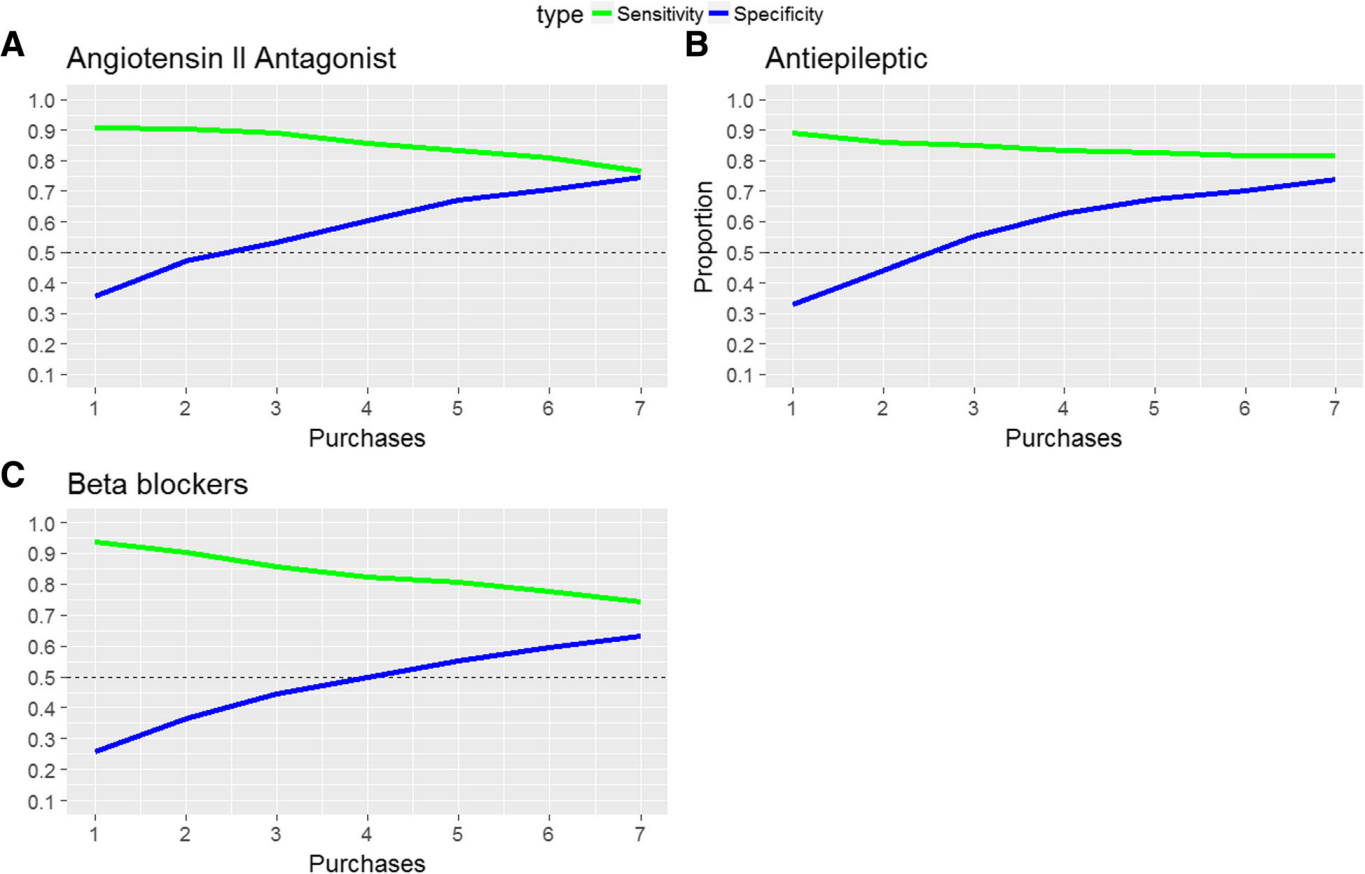
The sensitivity, specificity, and accuracy of predicting positive treatment response for prophylactic treatment. The figure presents the sensitivity (green), and specificity (blue) at different thresholds of minimum number of purchases to predict a positive treatment outcome. Letters referrers to the three different prophylactic treatments most commonly used, **a** Angiontensin II Antagonist, **b** Antiepileptic, & **c** Beta blockers

We identified an association between the number of purchases of prophylactic drug and a positive treatment effect (*p* < 1e16), see Fig. 3. The results did not change when excluding patients with comorbid epilepsy (*n* = 85) or hypertension (*n* = 473), although the average number of purchases dropped significantly for antiepileptics, see Additional file 1: Figure S4. To obtain a specificity above 50%, at least three purchases of angiotensin II antagonist and antiepileptics and four purchases for beta-blockers was needed (Fig. 4 - blue line). We observed that 68, 63, and 63% of the sample had purchased angiotensin II antagonist and antiepileptic three times and beta-blockers four times. The sensitivity is relatively high (> 80%) for four purchases or less.

## Discussion

Using a large clinical migraine cohort (*n* = 1913) including information on treatment response, we found a highly significant association between the number of purchases of triptans and positive treatment response, defined as having at least 50% reduction in symptoms within two hours of treatment start. We showed that the sensitivity was high (> 80%) for prediction of positive response to triptans and that the sensitivity was above 70% if the patients had more than ten purchases. We did not find evidence for an association between treatment response and purchases of non-migraine specific analgesics or ergotamines. We did not expect to observe an association, as non-migraine specific analgesics can be bought over-the-counter and are used for many other indications putatively introducing substantial statistical noise.

It is expected that migraine patients who purchase triptans more than once only do so if they experience a positive response. However, we observed an expected increase in sensitivity with number of purchases, although ten purchases of triptans were needed to reach 70% sensitivity. Since this is a retrospective study, this may be a consequence of recall bias. In addition, the positive response criterion of having 50% reduction of symptoms within two hours might exclude patients with marginally less or slower effect of triptans. An interesting future study would be to assess the individual migraine drugs. We demonstrated that analysing single drugs necessitates fewer purchases of the drug to gain the 80% specificity. However, a larger sample size is needed and, although more labour intensive, prospective studies may be needed. However, a key future goal is to compare treatment outcome with genetics, which requires that all participants must be genotyped. Here, thousands of patients are usually required to gain enough statistical power to assess common variants. Thus, a prospective migraine diary-based study seems less feasible.

We found a highly significant association between purchases of prophylactic drugs and a positive treatment response, defined as a more than a 50% reduction of their migraine attacks, from using angiotensin II antagonists, beta-blockers, or antiepileptics. We found a high sensitivity (> 80%) predicting positive treatment response from the number of purchases, and a specificity above 70% after at least three or four purchases depending on the drug. Whereas triptans are specific migraine drugs, the prophylactic drugs are not. As a result, the prediction model for prophylactic drugs necessitates knowledge of the prescription indication. Some drugs may be used to treat a multitude of conditions (e.g. epilepsy, arterial hypertension). Hence, we repeated the analysis excluding patients with comorbid disease. The association remained significant, although we observed a significant drop in the average number of purchases of antiepileptics.

Our study design included general triptan use and response, thus we were unable to test for an association between number of different triptans purchased and patients reporting no treatment effect. Future studies including more complex analysis of the types of triptans, patterns of medication exposures, etc., may aid in the characterization of migraine patient groups.

Precision medicine may offer new strategies to treat migraine, and access to existing large cohorts and pharmacy databases may help reach the required larger sample sizes. Based on the current results we recommend using ten purchases for triptans and three or four purchases for prophylactics. We anticipate that using fewer purchases, e.g. three triptans, may be sufficient; however, this should be evaluated in future studies. Notably, more than 75% of the triptan users have purchased triptans at least ten times, and 55–63% of the prophylactic users have at least three or four purchases. Additional factors may also influence treatment response, such as the use of other drugs [13–15], i.e. polypharmacy, as well as presence of co-morbid disorders. Here, it is possible that temporal aspect when additional drugs are prescribed, as a prescription could reflect treatment of adverse-effects. Further, it is easy to imagine that migraine patients with co-morbid depression may experience a better response to anti-depressants [16]. Most likely, genetic factors may also condition the treatment response, as seen for lithium and anti-psychotics [17–19], although studies on migraine drugs so far have been inconclusive or lack replication [13, 15].

## Conclusions

We conclude that a national pharmacy database is a valuable resource to identify a positive treatment response for migraineurs. As a general recommendation, we suggest using ten purchases for triptans and three or four purchases for prophylactic drugs, to predict positive treatment response. In future studies, more detailed information about treatment response and failure would improve the correlation with the number of purchases and perhaps enable us to predict treatment failure. We expect that a lower number of purchases than the ten suggested here may be sufficient.

## Additional file

Additional file 1: **Figure S1.** Distribution of prescription drugs for total acute treatment, Ergotamine and weak analgesic. **Figure S2.** Distribution of purchases given the number of different triptans purchased. **Figure S3.** Distribution and prediction for purchases for migraine patients without aura only. **Figure S4.** Distribution of prophylactic drugs with and without comorbid disoders. **Figure S5.** Receiver operating characteristic curve for model with and without gender and age as covariates. **Table S1.** Questions from semi structured interview. (DOCX 110 kb)

## Abbreviations

ACE: Angiotensin-converting-enzyme
ICHD: International Classification of Headache Disorders
OTC: Over-the-counter. This referrers to drugs that can be bought without a prescription
ROC: Receiver operating characteristics
