# Supplementary material for: Predicting treatment response using pharmacy register in migraine
Source: J Headache Pain. 2019 Apr 2;20(1):31. doi: 10.1186/s10194-019-0987-y (PMC6734320; doi:10.1186/s10194-019-0987-y)
Supplement: Supplementary file 1 — Figure S1. Distribution of prescription drugs for total acute treatment, Ergotamine and weak analgesic. Figure S2. Distribution of purchases given the number of different triptans purchased. Figure S3. Distribution and prediction for purchases for migraine patients without aura only. Figure S4. Distribution of prophylactic drugs with and without comorbid disoders. Figure S5. Receiver operating characteristic curve for model with and without gender and age as covariates. Table S1. Questions from semi structured interview. (DOCX 110 kb) [file 10194_2019_987_MOESM1_ESM.docx]

# Supplemental material

Suppl. figure 1. Distribution of prescription drugs for total acute treatment, Ergotamine and weak analgesic.


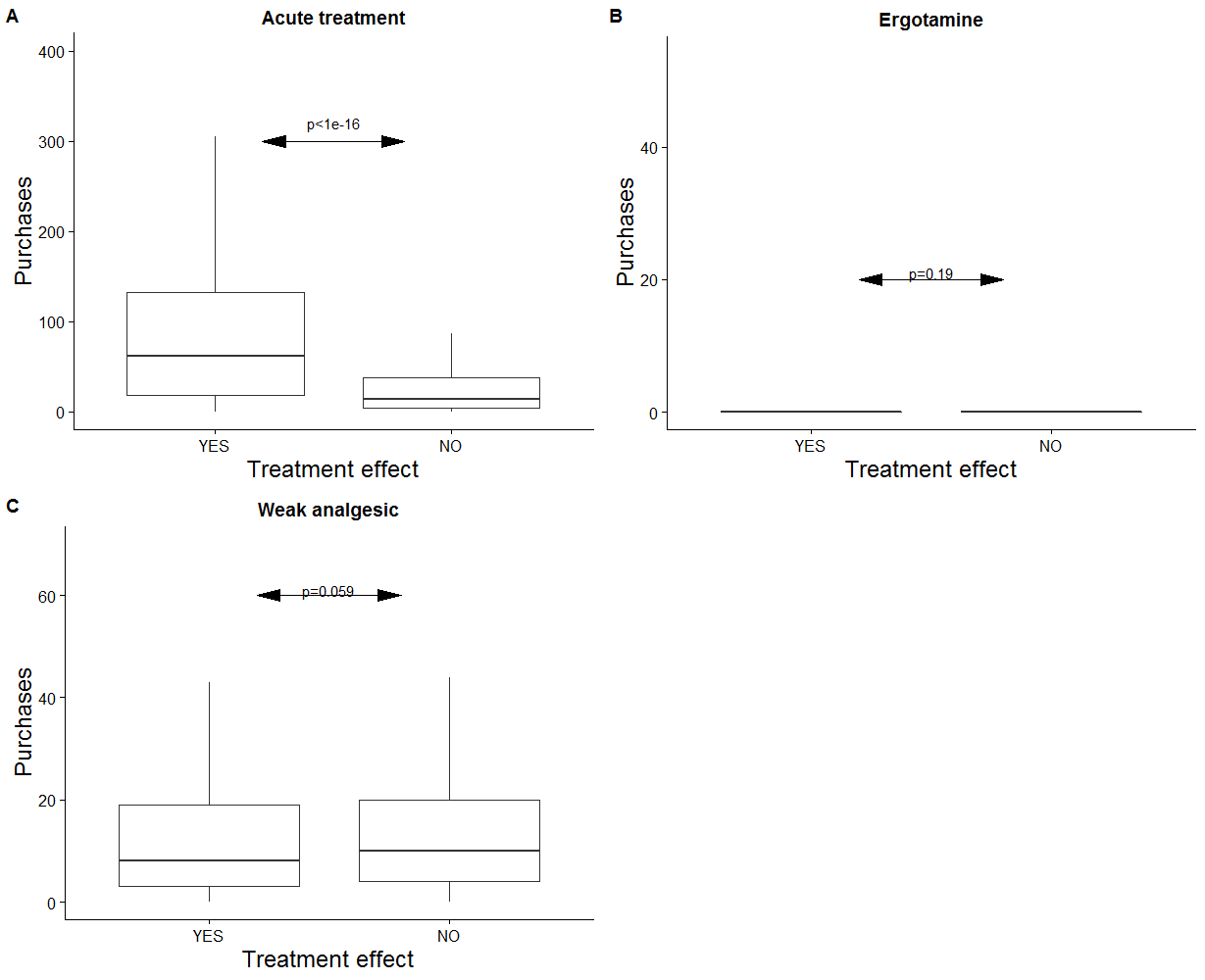


Suppl. Figure 2. Distribution of purchases given the number of different triptans purchased.


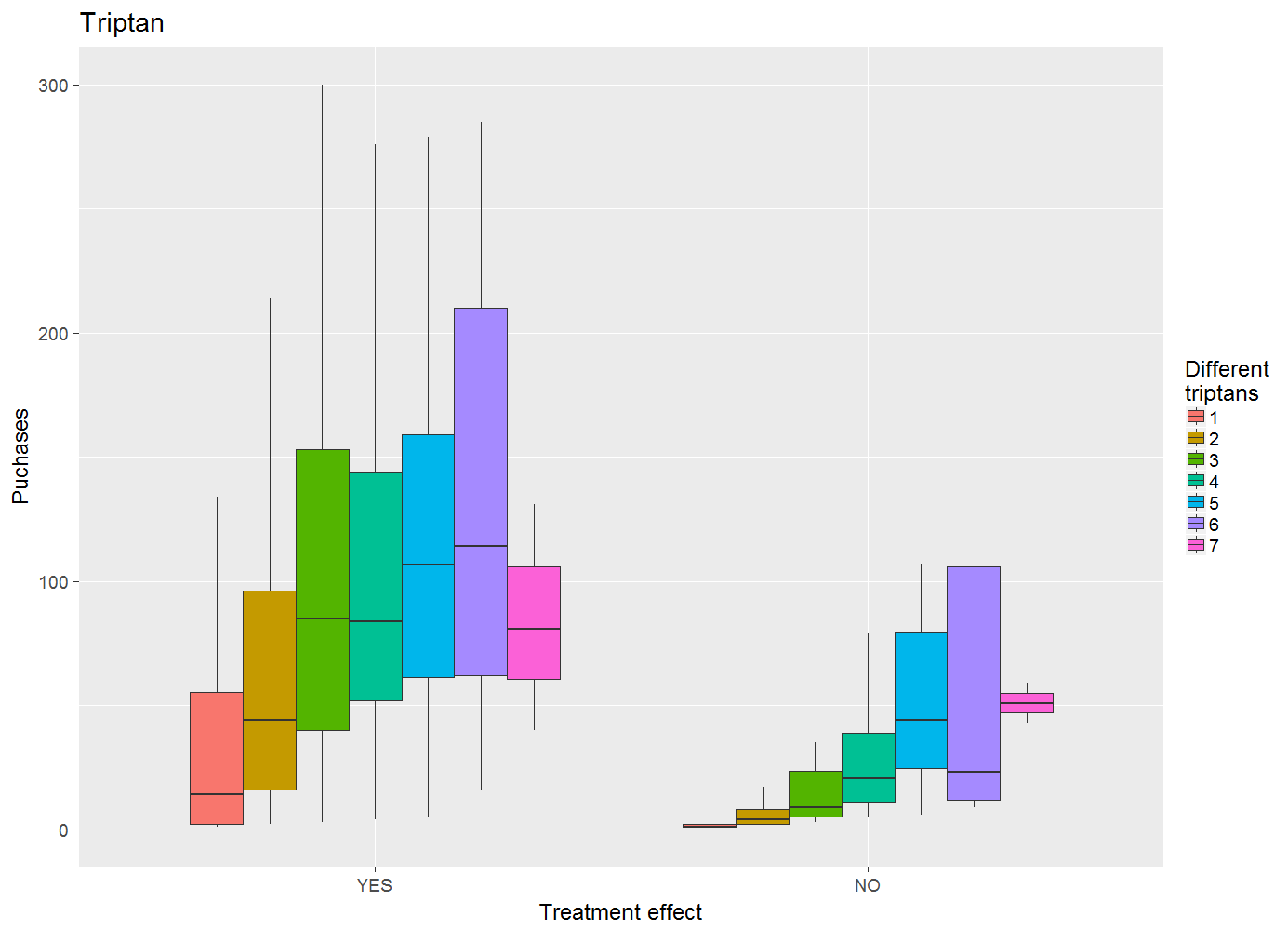


The distribution of prescription group by the number of different triptans prescribed(1-7), colored according to the legend on the right. The x-axis represents the reported treatment effect of triptans; Yes, No and Did not remember (DNR). Given the Danish legislation it is not allowed to depict individual data, thus, outliers are not shown.

Suppl. Figure 3. Distribution and prediction for purchases for migraine patients without aura only


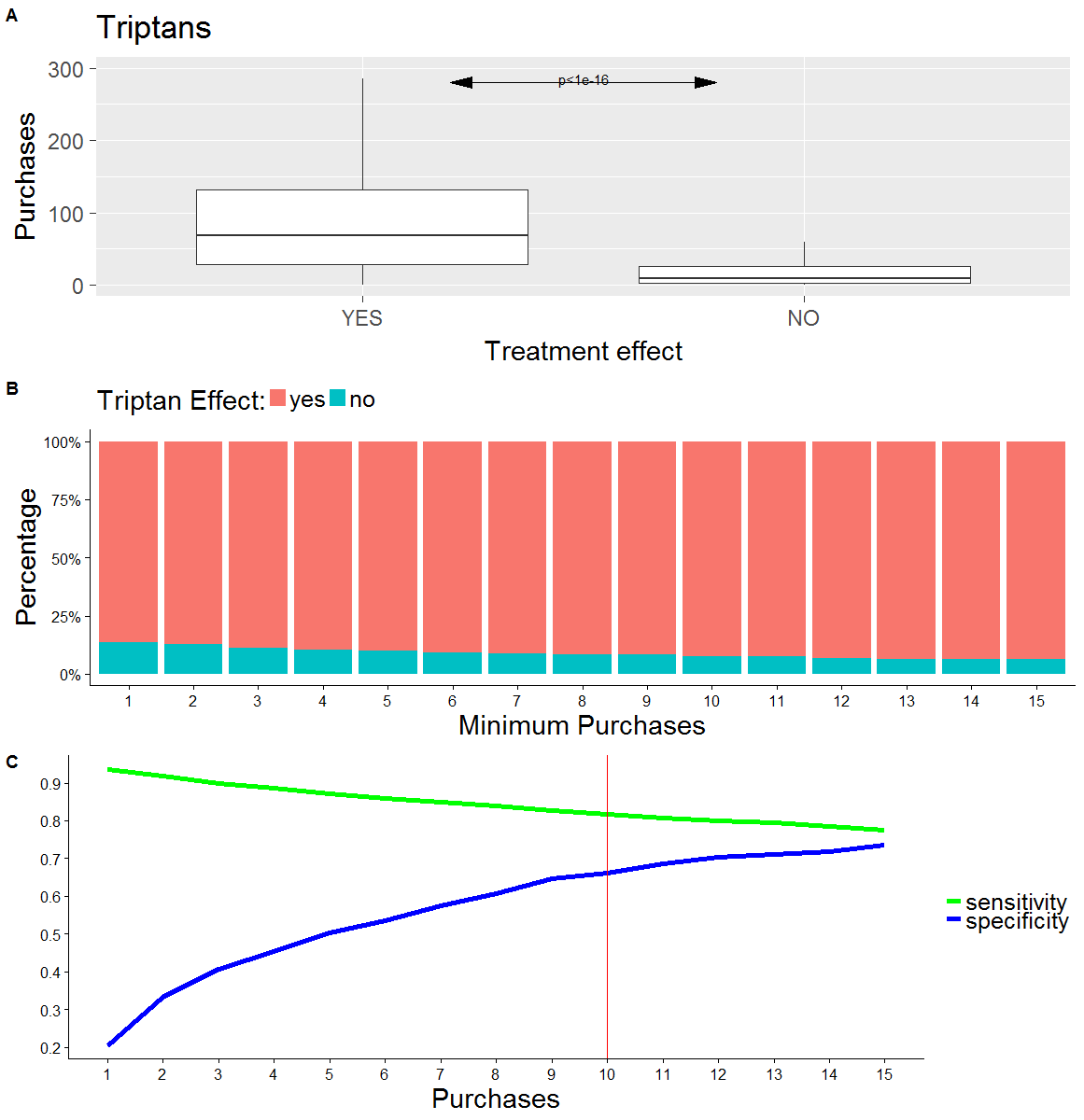


The figure includes only patients reporting migraine without aura. A; Distribution of number triptans purchased given treatment effect. B; The percentage distribution of treatment effect given the number of triptans purchased. C; The sensitivity(green) and specificity(blue) for prediction of positive treatment outcome of triptans given the number of triptans purchased.

Suppl. Figure 4. Distribution of prophylactic drugs with and without comorbid disoders


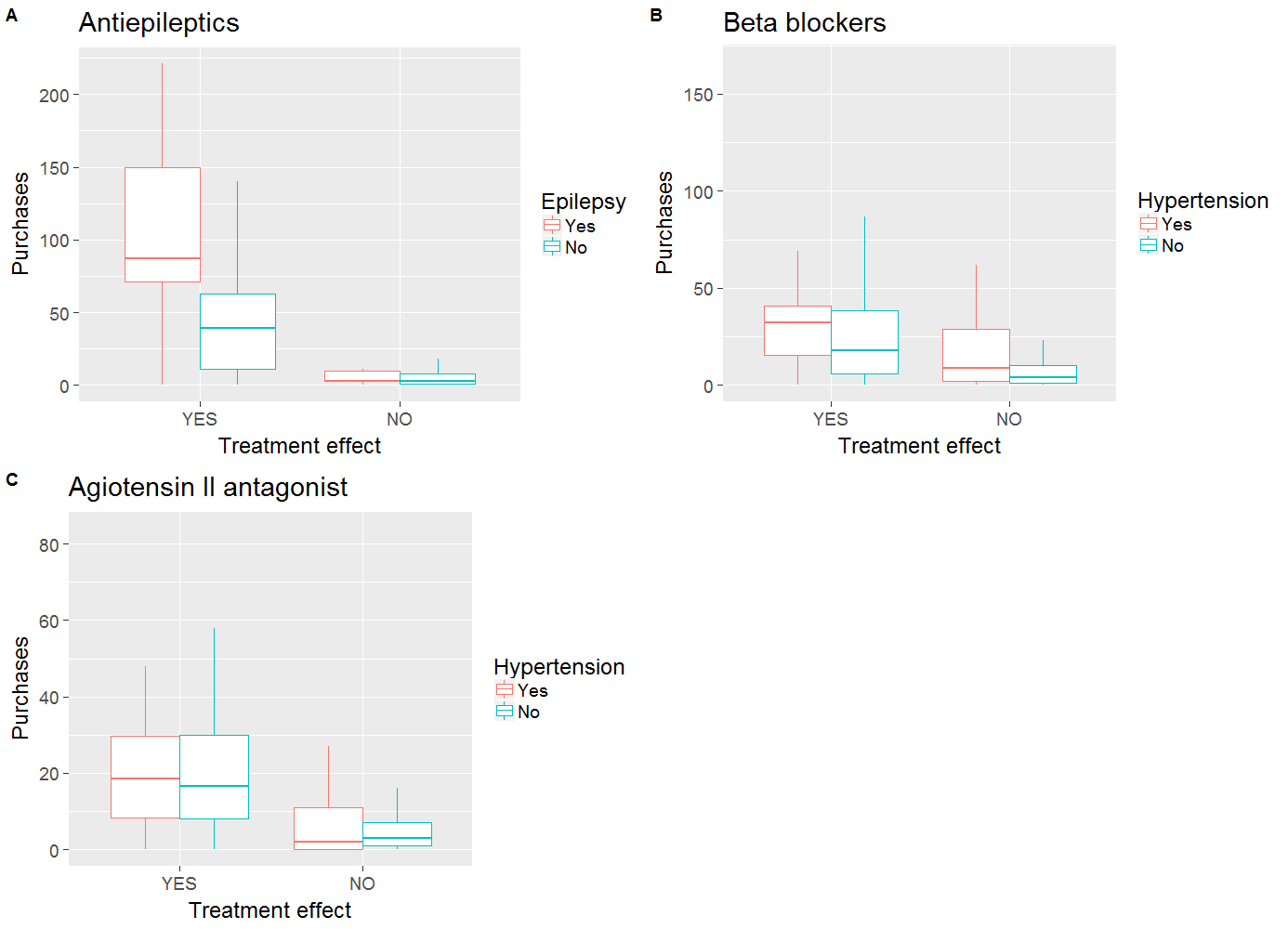


Distribution of prophylactic purchases for patients with and without comorbidity of A epilepsy and B & C Hypertension.

Suppl Figure 5, Receiver operating characteristic curve for model with and without gender and age as covariates


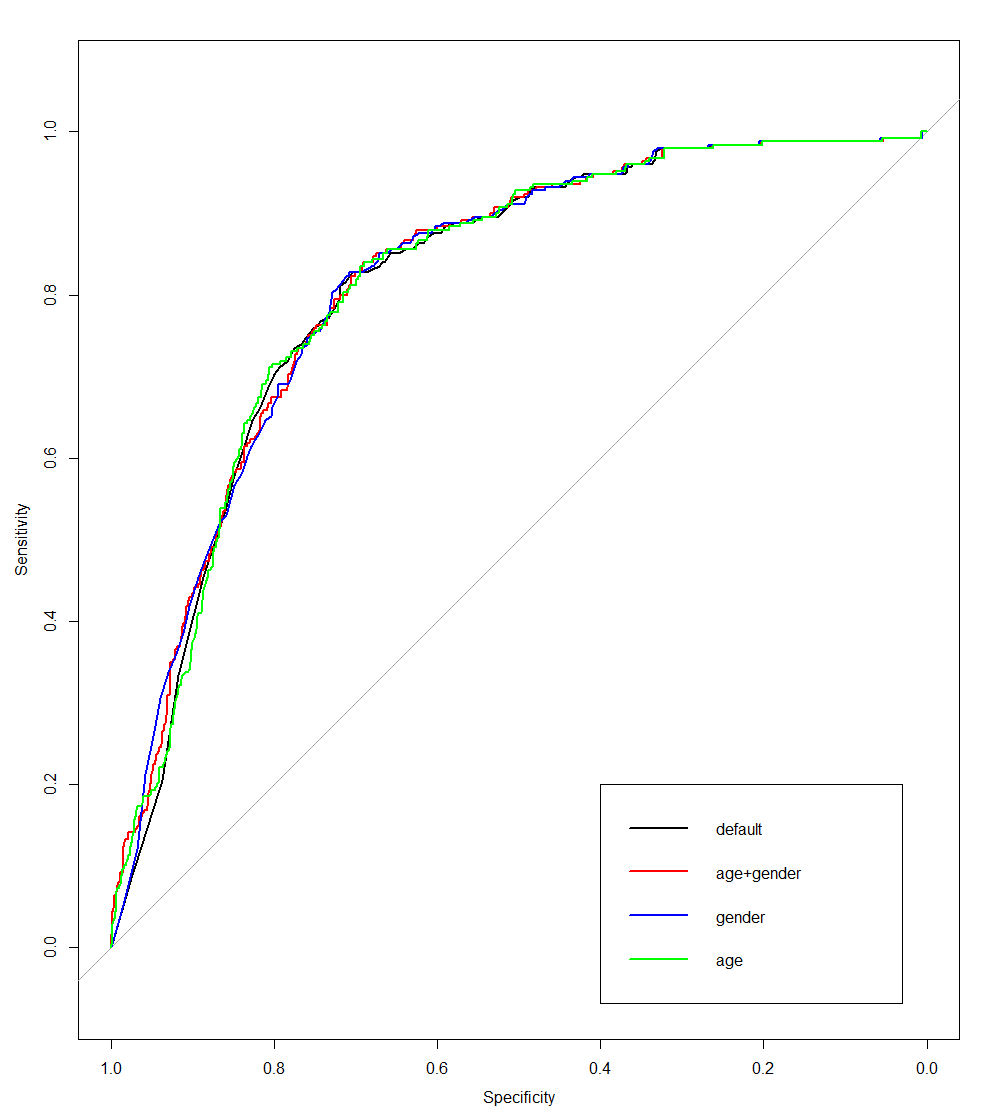


We use logistic regression with and without gender and age as covariates and in comparison, to default, i.e. with no covariates

**Suppl table 1, Questions from semi structured interview**

Usage of these question necessitate proper training and knowledge of headache disorders their pharmacological treatments. Only train medical doctors/students had performed interviews.

**Acute treatment Yes**  **No** Not relevant

Effect of triptan 1 2 3

Effect of weak analgesic 1 2 3

Effect of ergotamine 1 2 3

**Prophylactic treatment Yes** **No** Not relevant

Effect of β-blockers 1 2 3

Effect of Ca-antagonist 1 2 3

Effect of ang. II receptor antag. 1 2 3

Effect of antiepileptics 1 2 3

Effect of ACE-inhibitor 1 2 3

Effect of antidepressant 1 2 3
